# Self-harm in adolescence: protective health assets in the family, school and community

**DOI:** 10.1007/s00038-016-0900-2

**Published:** 2016-09-22

**Authors:** Ellen Klemera, Fiona M. Brooks, Kayleigh L. Chester, Josefine Magnusson, Neil Spencer

**Affiliations:** 1 The Centre for Research in Primary and Community Care (CRIPACC), University of Hertfordshire, College Lane Campus, Hatfield, AL10 9AL Hertfordshire UK; 2 Statistical Services and Consultancy Unit, Hertfordshire Business School, University of Hertfordshire, de Havilland Campus, Hatfield, AL10 9EU Hertfordshire UK; 3 Faculty of Health, University of Technology Sydney, PO Box 123 Broadway, Sydney NSW 2007, Australia

**Keywords:** Self-harm, Protective health assets, Family, School, Young people

## Abstract

**Objectives:**

The aim of this paper was to examine if the multiple environments of the adolescent including family, peers, school and neighbourhood might function as protective health assets against self-harming behaviour during adolescence.

**Methods:**

The present study utilised data collected from 1608 respondents aged 15 years as part of the England WHO Health Behaviour in School-aged Children (HBSC) Study. Multilevel modelling was undertaken using the package MLwiN (version 2.33) to investigate the potential domains and dimensions of family life, school culture and environment, and neighbourhood factors that may operate as protective health assets.

**Results:**

The results indicated that while peer support did not appear to operate as a protective health asset in the context of self-harm, key dimensions of adolescent/parent interaction and adolescent experience of the school culture and their neighbourhood were associated with reduced likelihood of self-harming behaviours during adolescence.

**Conclusions:**

The Findings highlight the significance of belonging and connectedness as important constituent elements of protective health assets for young people. Interventions that address the multiple environments of the young person, may offer an effective means to reduce the levels of self-harm.

## Introduction

Self-harm is commonly defined as the act of deliberately causing harm to oneself either by causing a physical injury, by putting oneself in dangerous situations, and/or self –neglect (Bifulco et al. 2014; Claes et al. 2015; http://www.nshn.co.uk/whatis.html).

Self-harm in adolescents is a major public health concern and one of the top five causes of hospital admittance in the UK (Burton 2014; Hawton et al. 2012; Mars et al. 2014; Shek and Yu 2012), with self-cutting appearing to be the most common method of self-harm in adolescents (Madge et al. 2008; Morey et al. 2016). Self-harm has been associated with depression, sleep problems, psychological distress and suicidal risk for adolescents (Burton 2014; Hysing et al. 2015; Kidger et al. 2012). Research indicates that young people who self-harm at age 16 are at increased risk of developing mental health and substance misuse problems, of self-harming in the future, and have a greater likelihood of inflicting suicidal self-harm (Burton 2014; Mars et al. 2014; Kidger et al. 2012; Kokkevi et al. 2012).

In the past two decades, research indicates that self-harm has become more prevalent among adolescents (Burton 2014; Hawton et al. 2012; Mars et al. 2014; Shek and Yu 2012; Kidger et al. 2012). Based on community studies from across the world around 13–18 % of adolescents experience a lifetime risk of self-harm (Mars et al. 2014; Kidger et al. 2012; Kokkevi et al. 2012; Landstedt and Gådin 2011). As many as one in fifteen young people self-harm in the UK, which is higher than the rest of Europe (Burton 2014). Despite the fact that only a small proportion of adolescents report self-harm to medical practitioners (one in eight, by Hawton et al. 2012), hospital statistics also show a dramatic increase in the prevalence of hospital admissions due to adolescent self-harm (Hawton et al. 2012).

Self-harm becomes increasingly common between the ages of 12 and 15 years, at which stage rates among adolescent girls are higher than boys (Burton 2014; Hawton et al. 2012; Shek and Yu 2012; Morey et al. 2016; Kidger et al. 2012; Kokkevi et al. 2012; Greydanus and Shek 2009; Spears et al. 2014). However, this pattern appears to change with age, recent research suggests that in the later teenage years self-harm is more prevalent among boys than girls (Hawton et al. 2012; Haast 2014). Although some authors regard self-harm rates more likely to be associated with lower socioeconomic groups (Hawton et al. 2012; Spears et al. 2014) this is contested and other studies have not identified any clear link between self-harm and a family’s economic status (Burton 2014; Shek and Yu 2012).

Low levels of family function (i.e. low levels of communication and high levels of conflict), a lack of parent–adolescent communication, and low levels of family cohesion and support have been associated with adolescent self-harm (Bifulco et al. 2014; Claes et al. 2015; Shek and Yu 2012; Greydanus and Shek 2009; Chandler 2014; Jablonska et al. 2009). Parental alienation (accompanied by intense parental criticism), family dysfunction, severe family neglect, intense conflict with peers, and especially being a victim of bullying have been found to be contributing factors to self-harm (Bifulco et al. 2014; Claes et al. 2015; Shek and Yu 2012).

There is also evidence that positive family communication and support, parental involvement, a caring neighbourhood and school climate, empowerment (e.g. from the community), family and school boundaries, and peer influence may work as protective against adolescent self-harm (Bifulco et al. 2014; Claes et al. 2015; Burton 2014). However, the existing deficit approach, while identifying the risk factors (or their absence) as influencing self-harm behaviour is not focused on the overall social environment as potentially protective for adolescent self-harm.

Although the above-mentioned investigations asserted the importance of family, school and neighbourhood for reducing of self-harm during adolescence, there are still very few studies (Shek and Yu 2012; Law and Shek 2013) that have tried to focus on the overall social environment around adolescence as protective of adolescents’ health in terms of self-harming behaviour. However, strengthening adolescents’ protective health assets (family and peer communication and support, caring neighbourhood and others) (Mannes et al. 2005) seem to be strongly associated with their psychosocial competence and therefore can result in protecting young people from self-harm (Claes et al. 2015; Shek and Yu 2012).

Moreover there has been relatively less work addressing the specific elements within health assets that might be operating as more protective than others, for example how might different aspects of parenting operate as determinants of adolescents’ health and well-being?

The aim of the current paper is to identify the elements of the multiple environments of the young person, i.e. Family, school, peers and neighbourhood that may potentially function as protective health assets in relation to the prevention of adolescents self-harming behaviour. In addition we seek to examine if different aspects within each of the environmental domains might be specifically operating as a protective health asset, for example, communication with parents and peers, sense of belonging to family, school or community, teacher connectedness and other social domains.

## Methods

### Procedure

The present study utilised data collected from 1608 respondents aged 15 years as part of the HBSC England 2013/2014 survey. The HBSC study is an international World Health Organization (WHO) collaborative study which explores the determinants of young people’s health and wellbeing, and health behaviours. The study collects data from school students aged 11, 13 and 15 years through anonymous self-completed questionnaires which young people complete during class time (Currie et al. 2010). Each country (currently, 42 countries are linked to the network) collect their own data, and in addition to a core mandatory set of questions countries can add their own questions of particular interest/relevance to that country. Questions on self-harm are not currently part of the mandatory questionnaire, but was added as a topic of importance in the questionnaire for England.

A random sample of all secondary schools in England (state and independent) stratified by region and school type was drawn. In total 48 schools were recruited, resulting in 5335 students from 261 classes. The final sample was representative of regional spread and school type. Response rate at the student level exceeded 90 %. Prior to the participation in the study, students and parents received information letters and an opt-out form if they did not wish to participate. For further details please see (Brooks et al. 2015a).

The study gained ethics approval via the University of Hertfordshire Ethics Committee for Health and Human Sciences (HSK/SF/UH/00007).

### Measures

Self-harm was measured by the question “have you ever deliberately hurt yourself in some way, such as cut or hit yourself on purpose or taken an overdose?” with response options yes and no.

The HBSC England 2014 questionnaire is a comprehensive measure of the health and wellbeing, health behaviours and social environment of young people. The following variables were included in the present analysis as they offered a way to examine protective factors as opposed to predictors of risk (such as bullying):

#### Family health assets

Assets relating to family life include parental communication with mother (FCM) and family communication with father (FCF) which ask respondents how easy it is for them to talk to their parents about things that bother them on a 4 point scale from “very easy” to “very difficult”. Responses were collapsed into a binary variable of “easy” vs “difficult”. A measure of family sense of belonging (FSB) was computed based on how often young people participated in shared activities with the family e.g. play sports together. Responses to items were summed with everyday = 5 and never = 1. FSB was categorised into low (4–8), medium (9–12) and high (13–20). Personal autonomy in relation to family (PAF) was measured by the question “how much say do you have when you and your parents are deciding how you should spend your free time outside school?” Response option “I usually decide” was categorised as high PAF, “my parents and I decide, but I usually do what I want” was categorised as medium PAF and “my parents usually decide” and “my parents and I decide, but I usually do what they want me to do” were both categorised as low PAF. Family social support (FSS) was measured through the Multidimensional Scale of Perceived Social Support (Zimet et al. 1988); responses to the four items concerning family support were averaged to provide an overall score of FSS.

#### School

School sense of belonging (SSB) and teacher social support (TSS) were included as school assets. A score for SSB was calculated by summing responses to the items “the students in my classes enjoying being together”, “I feel like I belong in this school” and “I feel safe in this school”; where strongly agree = 5 and strongly disagree = 1. Respondents overall score was then categorised into low (3–6), medium (7–11) and high (12–15) SSB. TSS was computed similarly using the items “I feel my teacher accepts me as I am”, “I feel that my teachers care about me as a person” and “I feel a lot of trust in my teachers”.

#### Peers

The multidimensional scale of perceived social support (Zimet et al. 1988) was used to measure peer social support (PSS); responses to the four items concerning peer support were averaged to provide an overall score of PSS.

#### Neighbourhood

The protective health asset ‘neighbourhood sense of belonging’ (NSB) was assessed via seven items including “people say hello and often stop to talk to each other in the street”, “it is safe for younger children to play outside during the day” and “I feel safe in the area where I live”. Responses to the items were summed with strongly agree = 5 and strongly disagree = 1, before being categorised into low (7–14), medium (15–27) and high (28–35) NSB.

Demographic variables including gender, age, ethnicity and family affluence were also included as potential explanatory variables. Family affluence was measured using the Family Affluence Scale,a measure of social economic status based on a set of six questions about the material conditions of the family home (Currie et al. 2014). Items are summed to produce a score between 0 and 13, and respondents were categorised into low (0–6), medium (7–10) and high (11–13) family affluence.

### Statistical methods

The self-harm outcome variable was a binomial variable (respondents answered “yes” or “no”). As the data was collected from groups of pupils in classes grouped into schools, multilevel modelling was undertaken using the package MLwiN (version 2.33) via the R2MLwiN package (version 0.8-0) in R (version 3.1.0).

The model building strategy that took place was to carry out a forward selection of main effects for the model from the list of potential explanatory variables. Wald tests were used to judge significance. The 1 % level of significance used rather than 5 % so as to allow for the fact that multiple hypothesis tests were being conducted. The inclusion of random slopes and then interactions between main effects were then considered using the 0.1 % level of significance so as to avoid the inclusion of spurious effects/interactions. At each stage, removal of terms from the model was considered.

## Results

### Sample characteristics

**Fig. 1:**
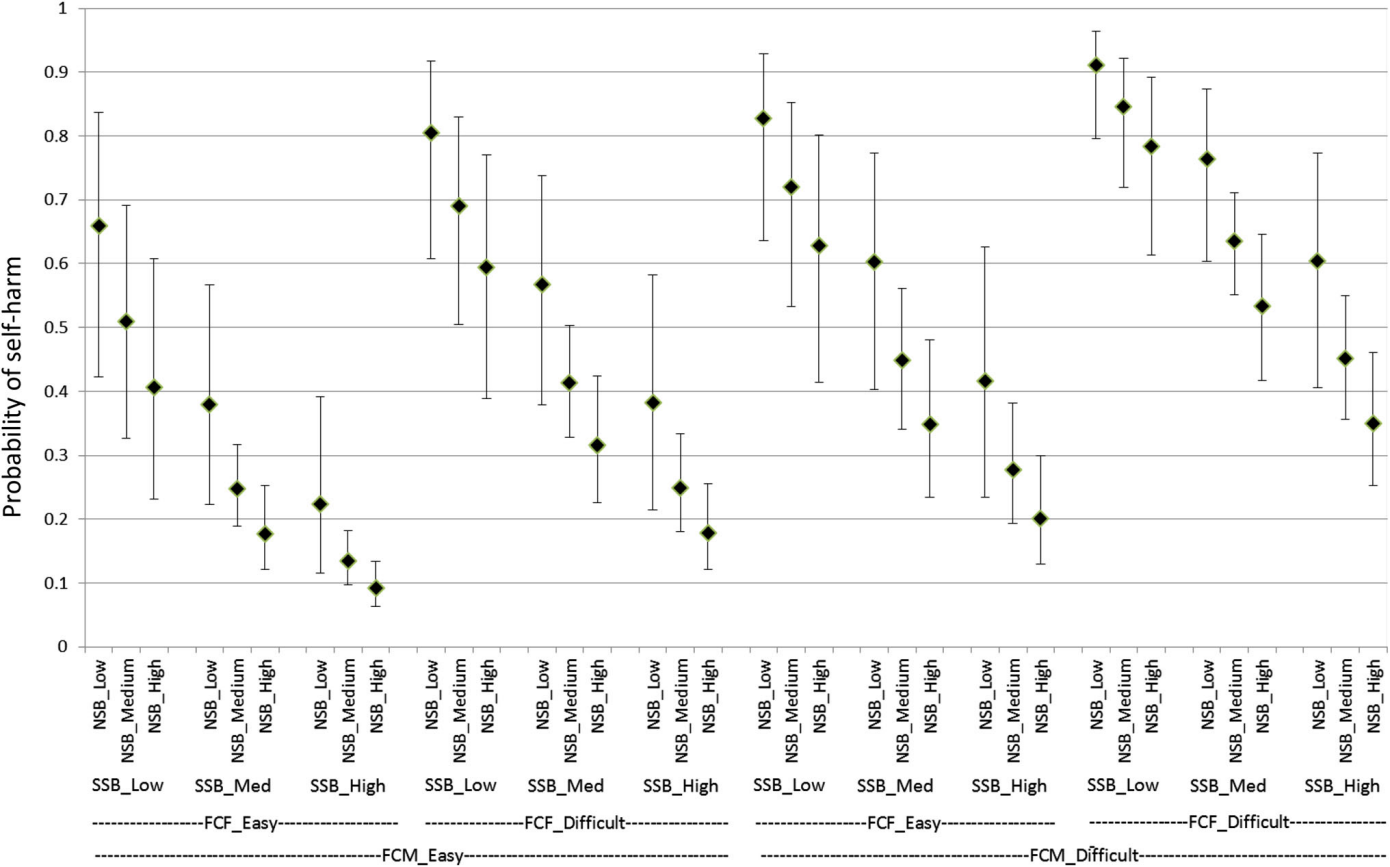
Estimated probability (with 95 % CI) of girls reporting self-harm for with varying combinations of explanatory variable. England, 2014.* CI* confidence interval,* FCM* communication with mother,* FCF* communication with father,* SSB* school sense of belonging,* NSB* neighbourhood sense of belonging

**Fig. 2:**
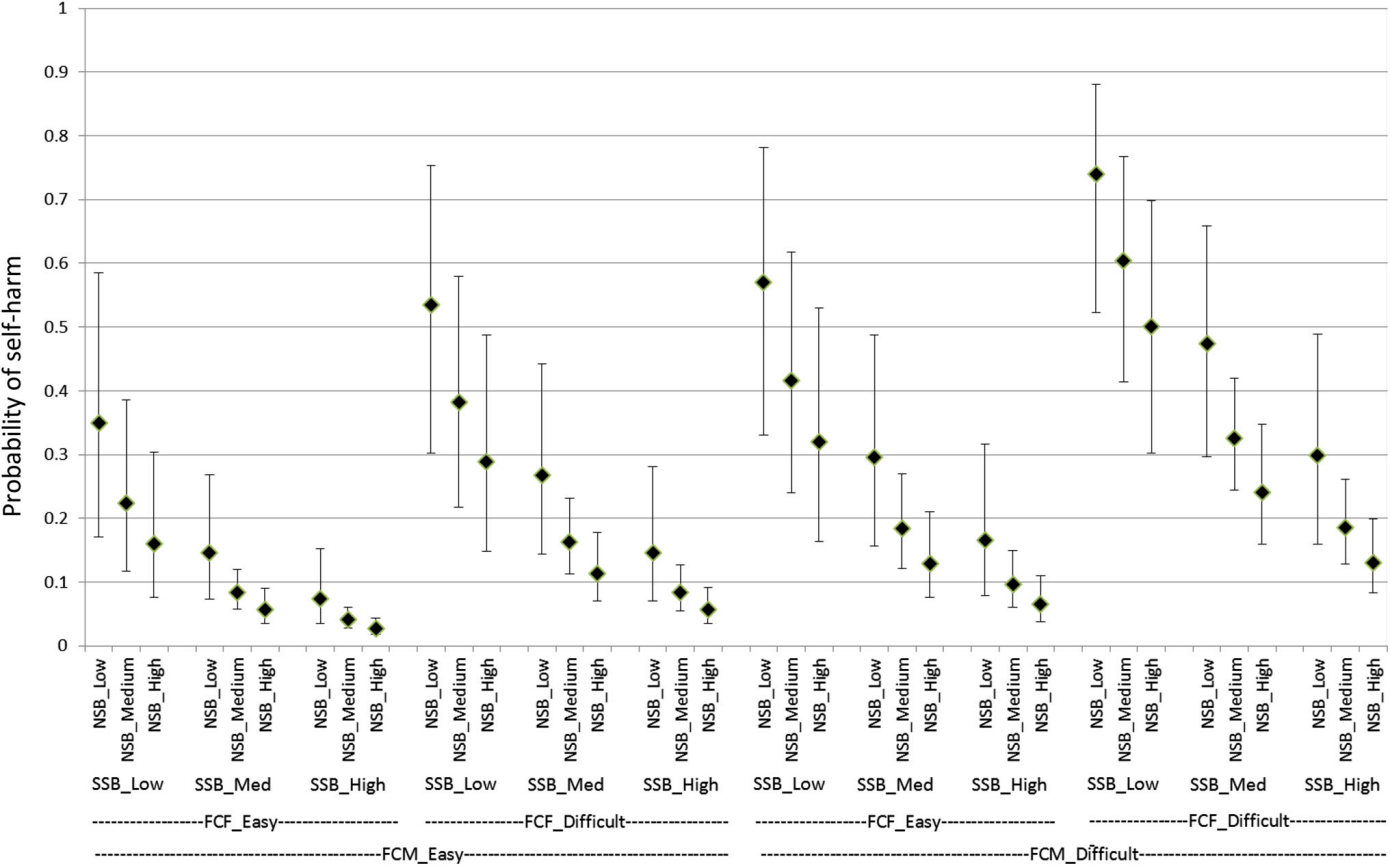
Estimated probability (with 95 % CI) of boys reporting self-harm for with varying combinations of explanatory variable. England, 2014.* CI* confidence interval,* FCM* communication with mother,* FCF* communication with father,* SSB* school sense of belonging,* NSB* neighbourhood sense of belonging

A total of 1608 15-year-olds completed the survey. 87 (5.4 %) participants omitted from answering the self-harm question, resulting in a sample of 1519 for the present study. Among the remaining participants, 823 (51.2 %) were boys and 784 (48.8 %) were girls. The vast majority of respondents reported being born in England (90.2 %), and over three quarters of the sample recorded their ethnicity as white (76.3 %). Having ever self-harmed was reported by 327 (21.5 %) out of 1519 young people. Girls were nearly three times as likely as boys to report ever self-harming (Table 1; Figs. 1, 2).

**Table 1:** Prevalence of reported self-harm, by gender. England, 2014

	*N*	%
Boys	88	11.4
Girls	239	31.9
Total	327	21.5

### Statistical model

A total of five variables were retained in the final model. No random slopes or interactions entered the model. Results are given in Table 2 as odds ratios (OR) with 95 % confidence intervals (CI) and *p*-values. Due to the multiplicity of comparisons that were conducted, results are only highlighted in table and discussed below where statistical significance reaches the 1 % level. Comparisons with a *p-*value of less than 0.01 have been highlighted in bold.

**Table 2:** Odds of reporting self-harming by explanatory variables, with confidence intervals (CI) and relevant *p* values. England, 2014

Variable	Comparison	OR	95 % CI	*p* value
Gender	Girls compared with boys	3.60	(2.56, 5.05)	<0.001
FCM	Difficult FCM compared with easy FCM	2.47	(2.32, 2.63)	<0.001
FCF	Difficult FCF compared with easy FCF	2.14	(2.02, 2.27)	<0.001
SSB	Low SSB compared with high SSB	6.70	(3.15, 14.25)	<0.001
	Low SSB compared with medium SSB	3.16	(1.52, 6.59)	0.002
	Medium SSB compared with high SSB	2.12	(1.53, 2.94)	<0.001
NSB	Low NSB compared with high NSB	2.84	(1.30, 6.19)	0.009
	Low NSB compared with medium NSB	1.86	(0.91, 3.83)	*0.091*
	Medium NSB compared with high NSB	1.52	(1.05, 2.21)	0.027

*CI* confidence interval, *FCM* communication with mother, *FCF* communication with father, *SSB* school sense of belonging, *NSB* neighbourhood sense of belonging

The main effects contained in the model were as follows:

#### Gender

Girls were estimated to have 3.60 times greater odds of reporting self-harm than boys (Table 2; Figs. 1, 2).

#### Communication with mother (FCM)

Those rating communication with their mother as “difficult” were estimated to have 2.47 times greater odds of reporting self-harm than those who rated their communication as “easy” (Table 2; Figs. 1, 2).

#### Communication with father (FCF)

Those rating communication with their father as “difficult” were estimated to have 2.14 times greater odds of reporting self-harm than those who rated their communication as “easy” (Table 2; Figs. 1, 2).

#### School sense of belonging (SSB)

Those with Low SSB are estimated to have approximately 6.70 times greater odds of reporting self-harm as those with High SSB and 3.16 times greater odds than those with Medium SSB. Those with Medium SSB are estimated to have approximately 2.12 times greater odds of reporting self-harm as those with High SSB. (Table 2; Figs. 1, 2).

#### Neighbourhood sense of belonging (NSB)

Those with Low NSB are estimated to have 2.84 times greater odds of reporting self-harm as those with High NSB. (Table 2; Figs. 1, 2).

## Discussion

The findings presented here aimed to employ an asset-based analysis to address self-harm, from the multiple aspects of the adolescents social life including family, school and community; and it is the first study of its kind (in a national English sample of 15 year old young people) to identify potential health assets that might operate as protective in relation to self-harming behaviour.

The present study, in line with other asset-based research conducted in the frame of HBSC data (Brooks et al. 2012; Fenton et al. 2010), identified associations between important external protective health assets that were located within the environment of the young person, and protection from self-harming behaviours.

The methodology used provided the possibility to test a large number of social contextual factors influencing self-harm in adolescence. The HBSC study is a unique cross-sectional survey that enables exploration of young people’s social environments as determinants of their health and well-being and health behaviours, providing a detailed picture of the whole social context in which young people live. Considering different aspects of adolescent’s life, the HBSC study provided the possibility for an overall asset-based analysis, including important parts of adolescent life such as family, school and community (Currie et al. 2012).

In line with previous research, this study found self-harm to be more prevalent among girls than boys in a sample of 15 year olds in England. If considered as a coping mechanism, this higher incidence among girls may reflect the pressures and poor emotional wellbeing that is reported more frequently among girls than boys in this age group (Brooks et al. 2015a).

This study highlighted the significance of connections with others and a sense of belonging has for adolescent health and well-being. Recently a growing body of work has identified the importance of connections with parents during adolescence for the maintenance of emotional wellbeing and health during adolescence (Brooks et al. 2015b; Cava et al. 2014; Levin et al. 2012; Rothon et al. 2012). The finding of an association between self-harm and positive communication with parents is in line with previous research that indicates that young people who reported self-harm have fewer people they can talk to about their problems (Evans et al. 2005). It is interesting that we found this effect to be significant only for people relating to the adult world; quality of communication with peers was not retained in the model and thus it appears that the protective relationships are primarily those with supportive adults. This highlights the limitations of a simplistic approach to adolescent social relationships and in particular the notion that peers naturally displace parents in the young person’s life as the main social support network. More recent research suggests both the continued importance of parental support during adolescence and a dynamic interaction between quality of parental relationships and peer relationships (Bifulco et al. 2014; Claes et al. 2015; Burton 2014) notably are an important element among those young people who self-harm. In particular this study highlights the importance of adult connections in the life of the adolescent.

From the results presented here young people’s sense of belonging and connectedness to school and the wider neighbourhood appears to be a strong health asset protecting from self-harm. This is in line with research which highlights the importance of adult connections for adolescents’ life; meaningful connections that support resilient or positive coping behaviours when difficulties arise seem to be crucial for adolescents. Research suggests that self-harm is a coping behaviour, but that it is located in internalising and avoidant strategies which are known to reinforce feelings of hopelessness and promote depression (Evans et al. 2005).

Existing research has indicated a strong association between a sense of belonging to school and wellbeing (Kia-Keating et al. 2011; Vieno et al. 2007). The findings presented here relating to neighbourhood and school sense of belonging both highlight the significance of connection to place for emotional well-being of young people. Also, considerations of how community or neighborhood initiatives may impact positively on the prevention of self-harming behaviors are required.

### Study limitations

The results of the study are based on young people self-reports of self-harming and their subjective perceptions of other social measures. The self-report nature of the data relies on young people’s perception of self-harm and therefore has a disadvantage compared to research conducted in clinical samples. Future research exploring protective assets would also benefit from identifying not only external health assets, but focusing on internal assets as well.

Another limitation of the study is that the methodology used (the multilevel analysis) identifies some associations between self-harm behaviour and various health assets in adolescents’ lives but it is not a predictive model as association does not necessarily mean causal relationships. So we can say that that there is an association between parental communication, belonging in neighbourhood and school, and ever self-harming in adolescence, but we cannot say if poor communication and poor sense of belonging in school and neighbourhood results in self-harming or vice versa. Further investigation is needed to gain more knowledge and understanding regarding causal relationships between health assets and self-harming in adolescence.

### Conclusions

Overall these findings highlight the significance of both belonging and connectedness as important constituent elements of protective health assets for young people. Having easy and open communication style as a parent appears to offer a protective element for young people even more so than interaction with peers, thereby highlighting that quality parenting is valuable for the promotion of adolescent well-being. The significance of feelings of belonging to school and neighbourhood highlight the importance for educational providers in establishing a positive ethos and culture within school settings but also that interventions across the environments of the adolescent should not only be located in educational setting or entirely focused on social learning interventions, instead attention needs also to be given as how community or neighborhood initiatives may impact positively on health and well-being for adolescents. Taken collectively the findings presented suggest that interventions adopting an ecological perspective across the multiple environments of the young person, including whole school approaches, may offer an effective means to reduce the levels of self-harm.
